# Factors Influencing the Acceptability and Uptake of HIV Self-Testing Among Priority Populations in Sub-Saharan Africa: A Scoping Review

**DOI:** 10.3389/phrs.2025.1608140

**Published:** 2025-04-22

**Authors:** Felix Emeka Anyiam, Maureen Nokuthula Sibiya, Olanrewaju Oladimeji

**Affiliations:** ^1^ Faculty of Health Sciences, Durban University of Technology, Durban, South Africa; ^2^ Vice-Chancellor and Principal’s Office, Mangosuthu University of Technology, Umlazi, South Africa; ^3^ Department of Public Health, Sefako Makgatho Health Sciences University, Pretoria, South Africa

**Keywords:** HIV self-testing, barriers, acceptability, priority populations, Sub-Saharan Africa

## Abstract

**Objectives:**

To identify and synthesize the factors influencing the acceptability and uptake of HIV self-testing (HIVST) among Priority Populations (PPs) in Sub-Saharan Africa (SSA) through a comprehensive scoping review.

**Methods:**

Using Arksey and O'Malley’s framework refined by Levac, we systematically reviewed the literature on factors affecting HIVST uptake and acceptability among PPs in SSA. The review included searches in six databases (Embase, Medline (via Ovid), PubMed, PsycINFO, Web of Science, WHO Global Health Library), as well as grey literature, including (Google Scholar and OpenGrey), limiting publications to 2010–2023.

**Results:**

The review found evidence indicating that HIVST is widely accepted and considered convenient among priority groups. Key challenges include limited post-test counseling and linkage to care, which hinder effective implementation. Peer-led and digital distribution strategies show the potential to increase uptake. However, user errors and economic constraints pose significant barriers to scaling HIVST, underscoring the need for targeted interventions to address these implementation challenges for optimal impact.

**Conclusion:**

While HIVST can boost testing rates among PPs in SSA, overcoming access and utilization barriers is crucial. Interventions addressing economic, educational, and systemic challenges are essential for successful HIVST integration into broader HIV prevention and care efforts.

## Introduction

The global HIV epidemic remains one of the most pressing public health challenges, with SSA bearing a disproportionate burden [1]. According to the UNAIDS 2024 Global HIV Statistics, an estimated 39.9 million people were living with HIV worldwide in 2023. Of these, 38.6 million were adults aged 15 years or older, and 1.4 million were children aged 0–14 years. Women and girls represented 53% of all people living with HIV [2]. In 2023, approximately 1.3 million people became newly infected with HIV, marking a 60% reduction in new infections since the peak in 1995. Despite this progress, the current rate of decline falls short of the target to reduce new infections to below 370,000 by 2025. AIDS-related illnesses claimed 630,000 lives in 2023, representing a 69% decrease since the peak in 2004 [2]. These statistics underscore both the progress made in the global HIV response and the challenges that remain in achieving the goals of ending AIDS as a public health threat by 2030.

Certain demographic groups in SSA face heightened HIV risks due to structural and social barriers rather than behavioral factors alone. Priority populations (PPs) are groups facing heightened HIV risks due to structural and economic barriers. These include young people, pregnant women, economically disadvantaged communities, individuals marginalized by sexual orientation or gender identity, and the partners of people living with HIV. Within this broader category, key populations (KPs) represent a subset experiencing higher HIV prevalence and systemic marginalization, such as men who have sex with men (MSM), female sex workers (FSWs), people who inject drugs (PWID), and transgender individuals [3]. Each group faces unique challenges: Adolescent girls face significant barriers to accessing sexual health services and education [4], while broader challenges also affect young people in general [5]. Stigma and legal obstacles have also been shown to significantly hinder key populations’ access to testing and care [6]. Socioeconomic barriers limit access to healthcare for low-income communities, while cultural and gender inequalities exacerbate HIV risks for women, restricting their autonomy in health-related decisions and access to HIV prevention and treatment services [7]. Globally, the median HIV prevalence among adults aged 15–49 is 0.8%, but priority populations experience significantly higher rates [2]. Among gay men and other MSM, prevalence is 7.7 times higher, while sex workers face HIV rates approximately 3 times higher than the general population. In eastern and southern Africa, young women and girls (15–24 years old) have an HIV prevalence 2.3 percentage points higher than the general population [2].

Effective HIV response strategies rely on accessible testing, yet systemic barriers prevent many priority populations from utilizing traditional services. Stigma, discrimination, lack of privacy, and fear of repercussions in healthcare settings often deter testing among pregnant women and their male partners as observed in the study by Naughton et al. [8]. HIVST addresses these challenges by offering a privacy-respecting testing method for individuals who might otherwise remain untested [9]. By enabling private self-testing, HIVST reduces stigma and fear of disclosure, facilitating entry into the HIV care continuum [10]. Evidence indicates that HIVST can boost testing uptake and early diagnosis among priority populations, including young okey populations such as MSM and FSWs, supporting timely linkage to care, addressing psychological and emotional barriers such as fear, denial, and anxiety, and promoting preventive practices [11, 12]. With UNAIDS’ 95–95–95 targets for 2030—95% of people living with HIV knowing their status, 95% of those diagnosed receiving sustained therapy, and 95% achieving viral suppression [1]—HIVST could be pivotal in SSA’s HIV response.

While HIVST shows promise, its acceptance and uptake among SSA’s priority populations require addressing regional and demographic barriers, including cost-related barriers, which may impact accessibility and affordability for certain populations [13], gender dynamics [14], stigma, and inconsistent healthcare infrastructure [12]. Understanding these obstacles is essential to improve access and willingness to use HIVST in these communities. This scoping review synthesizes evidence on the acceptability and uptake factors for HIVST among SSA’s priority populations, aiming to inform effective and tailored intervention design. By identifying both enabling factors and barriers, this review seeks to provide policy recommendations to optimize HIV testing coverage, supporting progress toward UNAIDS 2030 goals and advancing epidemic control.

## Methods

This scoping review follows Arksey and O'Malley’s methodology [15], refined by Levac et al. [16] through six steps: 1) identifying the research question, 2) selecting relevant studies, 3) selecting eligible studies, 4) charting the data, 5) collating, summarizing, and reporting findings, and 6) consultation. Our approach incorporates Joanna Briggs Institute principles [17], ensuring methodological rigor in identifying and synthesizing relevant literature.

A detailed protocol outlining the search strategy, inclusion criteria, and preliminary analysis plan has been published [18], supporting transparency and replicability. Additionally, we adhered to the PRISMA-P guidelines for procedure development [19] while the PRISMA-ScR extension guided the structuring and reporting of our findings [20].

### Stage 1: Identifying the Research Question

Following the approach recommended by Arksey and O'Malley, we formulated the primary research question: “What are the factors influencing the acceptability and uptake of HIV self-testing among PPs in SSA?”

The research sub-questions are:

i. What economic, social, and behavioral factors influence the acceptability and uptake of HIV self-testing among priority populations in SSA?
ii. What is the acceptability rate of HIV self-testing among priority populations in SSA?

For clarity, we define PPs and distinguish them from KPs to ensure consistency in terminology throughout the review. Priority populations encompass groups that experience heightened HIV risk due to structural and economic barriers, while key populations form a subset with disproportionately high HIV prevalence and systemic marginalization. These definitions are summarized in Table 1.

**TABLE 1:** Definitions of priority and key populations (Sub-Saharan Africa, 2010–2023).

Term	Definition
Priority Populations (PPs)	Groups facing heightened HIV risks due to structural and economic barriers, including young people, pregnant women, economically disadvantaged communities, and those marginalized by sexual orientation or gender identity
Key Populations (KPs)	A subset of priority populations who experience higher HIV prevalence and systemic marginalization, including MSM, FSWs, PWID, and transgender individuals

This study used the PIOT framework (Table 2) to align study selection with the research topic. In this PIOT (Population, Intervention/Exposure, Outcome, Timeline) format, the Population focuses on priority populations (PPs) in SSA, while the Intervention/Exposure is HIVST. The Outcomes assessed are factors influencing the acceptability and uptake of HIVST among these populations. This review includes studies published between 2010 and 2023 to capture recent developments in HIVST implementation and adoption. While some included studies specify whether they assessed oral-fluid or blood-based HIVST and whether testing was supervised or unsupervised, many do not provide these details. Given this variability, our review focuses on general findings regarding HIVST acceptability and uptake, without distinguishing between specific self-testing modalities.

**TABLE 2:** Population–intervention–outcome–timeline framework (Sub-Saharan Africa, 2010–2023).

Criteria	Determinants
P-Population	Priority Populations (PPs) in SSA
I-Intervention/Exposure	HIV self-testing, including both oral-fluid and blood-based methods. The review considers both supervised and unsupervised self-testing approaches
O-Outcomes	Acceptability and uptake of HIVST
T-Timeline	2010–2023

In this review, we define:

*Acceptability* as the willingness of individuals to use HIV self-testing, recommend it to others, or express a positive attitude toward it when offered [21].

*Acceptability rate* as the proportion of individuals who are willing to use, or have a positive attitude toward HIVST.

*Uptake* as the proportion of individuals who have actually used an HIV self-test kit, regardless of whether they reported their results or received post-test counselling [22].

### Stage 2: Identifying Relevant Studies

To identify relevant studies, we systematically searched electronic databases, including Embase, Medline (via Ovid), PubMed, PsycINFO, Web of Science, WHO Global Health Library and grey literature sources like Google Scholar and OpenGrey, covering publications from 2010 to 2023. The latest search was on 5 June 2024 (see Supplementary File S1 - Search Strategy for PubMed Medline). We also accessed dissertations via ProQuest. Our search combined keywords related to HIV, self-testing, priority groups, and SSA. Reference lists of included studies were reviewed to capture additional relevant studies.

### Stage 3: Study Selection of Eligible Studies

For systematic study selection, we used the PIOT framework (see Table 2) to guide title and abstract screening. Additional eligibility criteria were applied to further refine the selection, ensuring the inclusion of only studies directly relevant to our research question.

#### Inclusion Criteria

Two independent reviewers assessed the eligibility of titles and abstracts based on the specified inclusion criteria:

(1) The study was conducted in SSA
(2) Primary research articles (quantitative, qualitative, or mixed-methods studies).
(3) Government or organizational reports that include empirical data.
(4) The study focuses on priority populations, including adolescents, pregnant women, men, key populations such as MSM and FSWs, and other vulnerable populations.
(5) The study examines factors influencing the acceptability and uptake of HIVST.
(6) The study was published in English between 2010 and 2023.

#### Exclusion Criteria

Studies were excluded if they met any of the following criteria:

(1) Studies not conducted in SSA or studies that did not include participants from SSA.
(2) Studies where full-text articles were unavailable for review.
(3) Studies that did not focus on priority populations.
(4) Studies that did not examine factors influencing the acceptability and uptake of HIVST.
(5) Editorials, opinion papers, conference abstracts, and commentaries were excluded due to a lack of primary data.
(6) Studies published in languages other than English or published outside the timeframe of 2010–2023.

### Stage 4: Charting the Data

Guided by the PIOT framework, we charted and analyzed data from selected studies. Using a predefined charting form (see Table 3), we systematically recorded key information, including study design, setting, population, and major findings.

**TABLE 3:** Data charting form (sub-Saharan Africa, 2010–2023).

1	Lead author
2	Year of publication
3	Title of study
4	Aim of study
5	Study design
6	Study setting/country
7	Study Population
8	Age group
9	Sample Size (Number of participants)
10	Eligibility criteria
11	Intervention
12	Study Outcome/Result
13	HIVST Acceptability
14	Acceptability Rate
15	HIVST Uptake
16	Recommendations from the study
17	Main Findings

### Stage 5: Collating, Summarizing, and Reporting the Results

To analyze data from diverse study types in this review, thematic content analysis was used for qualitative studies to identify themes related to factors influencing HIVST acceptability and uptake, incorporating key quotes or narratives for insights into participant perspectives. Two independent reviewers screened titles and abstracts based on inclusion criteria, assessing full texts for final eligibility. Reviewer discrepancies in study selection and data extraction were resolved through discussion or a third reviewer. This ensured consistent, unbiased study selection and data extraction, using the Data Charting Form to align with the research objectives and study aims.

### Stage 6: Consultation

This scoping review was completed without stakeholder consultations. However, future engagement with key stakeholders—including public health officials, HIV program implementers, and community representatives—could further validate and contextualize the findings. Stakeholder insights would be valuable in refining implementation strategies, addressing barriers to HIV self-testing uptake, and guiding policy recommendations in SSA.

### Ethics and Dissemination

This scoping review of existing literature did not require ethical approval. Our dissemination strategy includes reaching both academic and community audiences. We plan to present findings through community meetings and workshops with local health departments and community organizations.

## Results

### Study Selection and Characteristics

**FIGURE 1:**
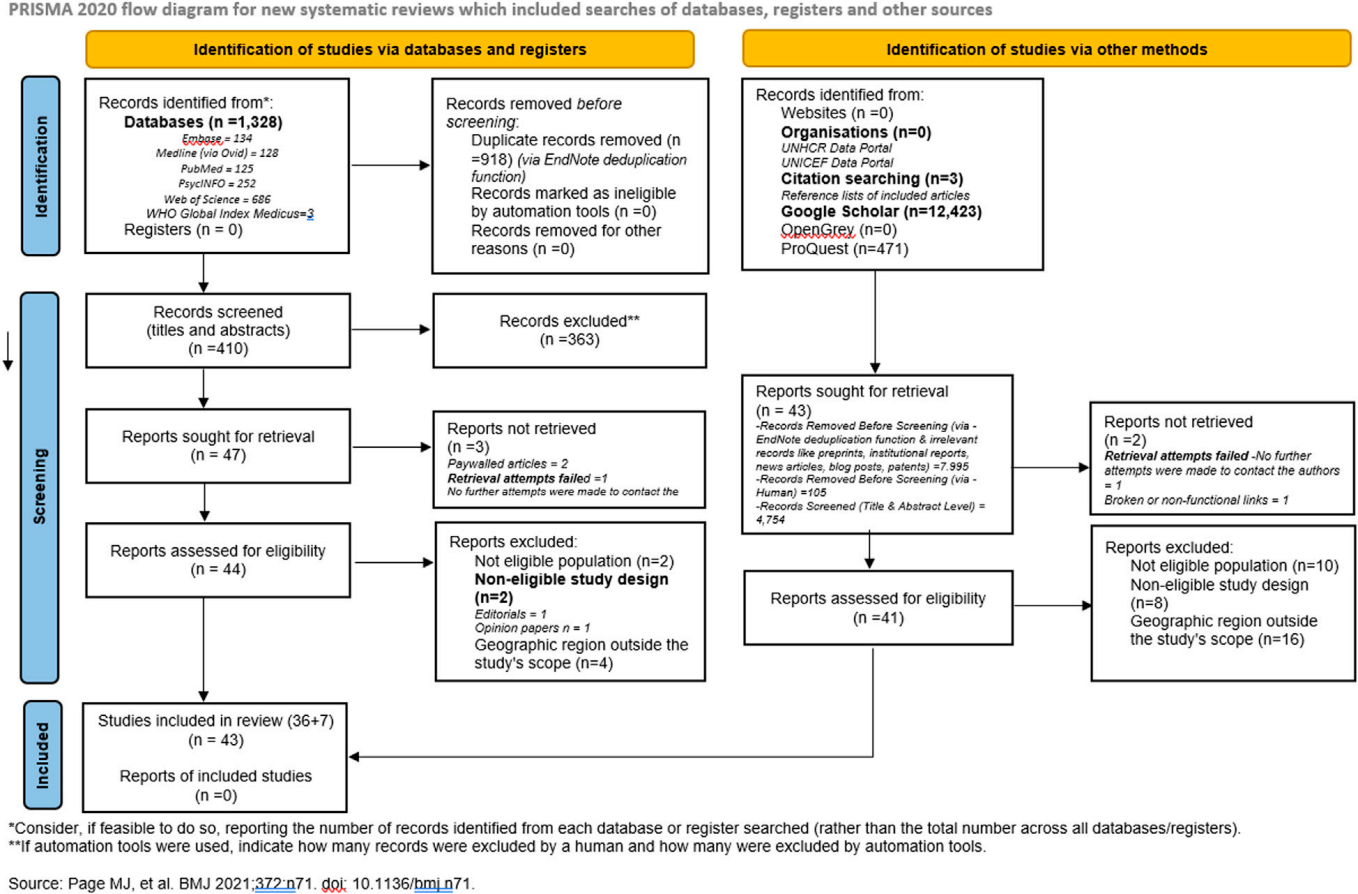
Preferred Reporting Items for Systematic Reviews and Meta-Analyses flow diagram summarizing search process and source selection (Sub-Saharan Africa, 2010 - 2023).

The search strategy identified 14,225 records, including 1,328 from databases (134 from Embase, 128 from Medline (Ovid), 125 from PubMed, 252 from PsycINFO, 686 from Web of Science, and 3 from WHO Global Index Medicus) and 12,897 from other sources, including Google Scholar (12,423), citation searching (3), and ProQuest (471). After the initial screening, duplicate records (n = 9,018) were removed using EndNote’s deduplication function (918 from databases and 7,995 from other sources), with an additional 105 manually removed. A further 5,117 records were excluded based on title and abstract screening (363 from databases and 4,754 from other sources), leaving 90 records for full-text retrieval (47 from databases and 43 from grey literature). Of these, 5 reports could not be retrieved due to paywalled access (n = 2), failed retrieval attempts due to non-responsive authors (n = 2), and broken/non-functional links (n = 1). This left 85 full-text reports assessed for eligibility (44 from databases and 41 from grey literature). Following the eligibility assessment, 42 records were excluded for the following reasons: Not eligible population (n = 12), Non-eligible study design (n = 10) (e.g., editorials, opinion papers), and Geographic region outside the study’s scope (n = 20). Ultimately, 43 records were included in the final review (36 from databases and 7 from grey literature). The selection process is outlined in Figure 1 (PRISMA flow diagram).

### Characteristics of Included Studies and Geographical Distribution

**FIGURE 2:**
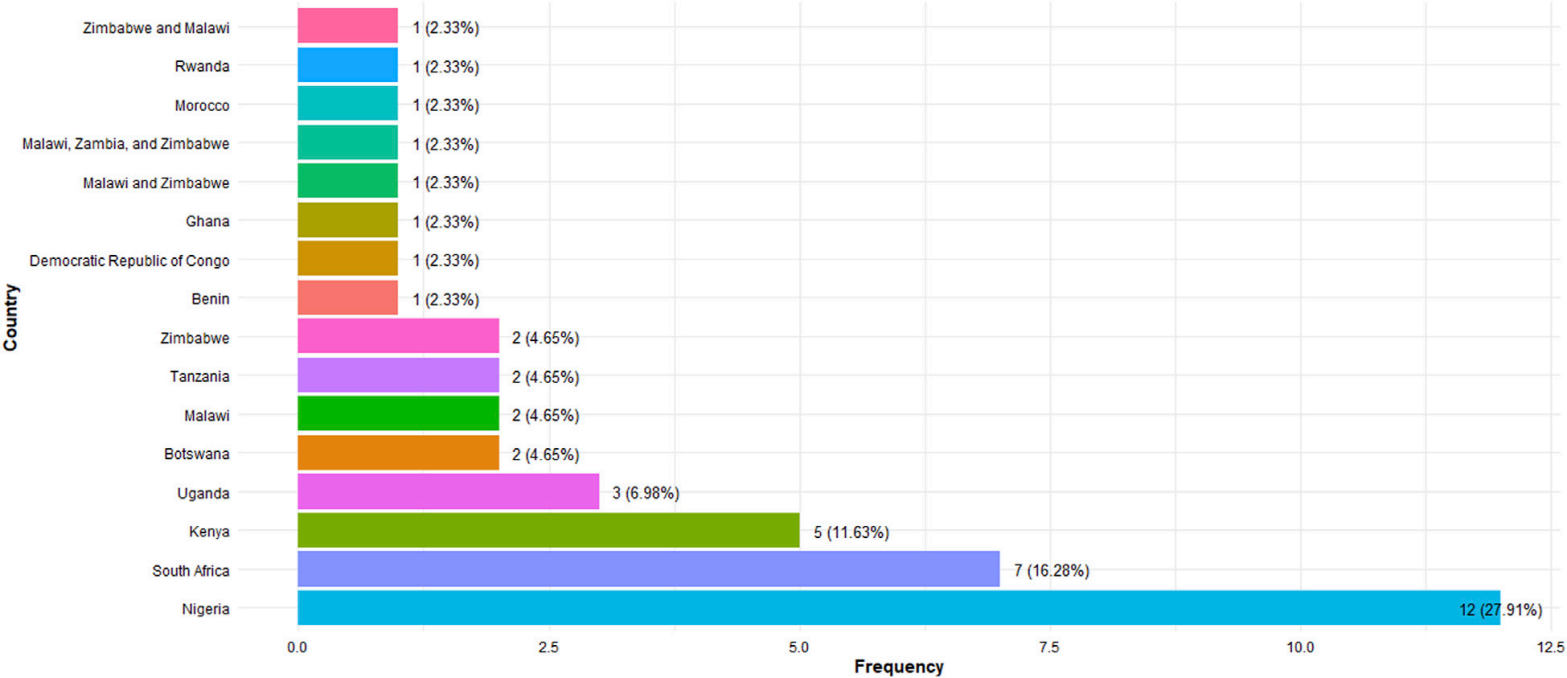
Countries of eligible studies (Sub-Saharan Africa, 2010–2023).

The 43 sources that met the inclusion criteria were published between 2010 and 2023. A summary of the main findings, including study characteristics, HIVST acceptability, uptake, and key recommendations, is provided in the Supplementary File S2: Table of Study Extraction. The different study designs were a Cross-sectional study (n = 17) [13, 23–38], Qualitative study (n = 16) [8, 39–53], Mixed methods study (n = 7) [54–60], Longitudinal study (n = 1) [61], Cohort study (n = 1) [62], and Prospective validation study (n = 1) [63]. Majority of the sources originated from Nigeria (n = 12), followed by South Africa (n = 7), Kenya (n = 5), Uganda (n = 3), Botswana (n = 2), Malawi (n = 2) and Tanzania (n = 2). The full list of countries is displayed in Figure 2. The reviewed studies spanned several countries within SSA, including Ghana, Nigeria, Malawi, Zimbabwe, Uganda, Kenya, Benin, and the Democratic Republic of Congo, encapsulating a broad geographical representation.

### Methodological Diversity in HIVST Research

Our scoping review included diverse methodologies to explore HIVST acceptance and implementation in SSA, ranging from descriptive analyses of community-based interventions in Ghana [23] to cross-sectional surveys targeting specific demographics in Nigeria [24, 31, 32], Malawi, Zambia, Zimbabwe [34, 36], Rwanda [35] and South Africa [38]. We also reviewed in-depth qualitative studies offering narrative insights on individual and community views on HIVST, such as those conducted in Uganda and Tanzania [8, 39, 46]. Mixed-methods studies, combining quantitative and qualitative data, provided a detailed examination of factors influencing HIVST acceptability in Nigeria, South Africa, Kenya, Malawi, and Zimbabwe [54–58]. This methodological diversity highlights the complexity of HIVST research and the value of multiple approaches to fully understand HIVST adoption.

### Statistical Approaches in Original Studies

Our analysis of the included studies revealed various statistical approaches used to evaluate HIVST acceptability in SSA populations.

Most cross-sectional studies applied descriptive statistics (e.g., means, medians, frequencies) to summarize participant demographics [25, 31, 33–35, 37]. Inferential statistics, like chi-square tests and logistic regression, were commonly used to identify significant factors associated with HIVST uptake, with logistic regression pinpointing demographic predictors of acceptability.

Mixed-methods studies combined thematic analyses with descriptive statistics, uncovering contextual factors such as stigma and privacy concerns that shape HIVST attitudes [49, 54–56, 58]. This integration enriched insights into HIVST adoption across populations by showing how personal and community factors intersect.

Though fewer, longitudinal studies employed repeated-measures analyses to track changes in HIVST attitudes over time [61, 62]. These studies provided a dynamic view of acceptability, showing how shifts in policy or socio-economic conditions influence HIVST sustainability and identifying groups needing continued support.

### Demographic Distribution and Population-specific Insights

This review included studies on diverse priority populations in SSA, vulnerable to HIV, covering MSM (10 studies) [13, 23, 27, 38, 44, 47, 54, 55, 61, 62], FSWs (8 studies) [26, 39, 41, 43, 45, 51, 53, 60], adolescents (13 studies) [25, 28–30, 33, 36, 37, 40, 42, 49, 50, 58, 59], pregnant women (2 studies) [8, 24] and general adult populations (10 studies) [31, 32, 34, 35, 46, 48, 52, 56, 57, 63]. FSWs and young adults showed high interest in HIVST due to its privacy [25, 42, 45, 53]. MSM and general adult populations had varied responses, influenced by factors like stigma and healthcare access [34, 38, 47, 52].

### High Acceptability Among Young Adults and FSWs

Studies show high acceptability of HIVST among young adults and FSWs, with reported rates ranging from 70% to 90% [37, 45, 50]. McHugh et al. [25] and Koris et al. [50] found that young adults, especially in informal urban areas, appreciated the privacy and autonomy HIVST offers, avoiding the stigma linked to traditional testing centers. Similarly, Shava et al. [45] and Boisvert Moreau et al. [53] reported strong acceptance among FSWs, who valued the ability to bypass public healthcare settings, where stigma and discrimination are concerns.

### Varied Acceptance Among MSM

HIVST acceptability among MSM populations varies, shaped by privacy concerns, stigma, and perceived discrimination. Dirisu et al. [47] found that stigma often deters MSM from traditional testing, making HIVST an appealing, less-exposed option, though privacy concerns remain, due to fears of being identified when collecting test kits or seeking post-test support. Also, while HIVST offers privacy, supervised self-testing approaches (where individuals are observed while conducting the test) raised privacy concerns. MSM feared counselors or community health workers might disclose their status to others, particularly in cases where known members of the MSM community facilitated testing. Knox et al. [38] observed that acceptance is higher among MSM in supportive or urban environments than in conservative areas, indicating that healthcare access and societal attitudes impact uptake. These findings suggest that targeted outreach and culturally sensitive education are crucial to addressing the complex barriers to HIVST adoption among MSM.

### Moderate Acceptability Among General Adult Populations

General adult populations showed moderate acceptability for HIVST, influenced by local HIV attitudes and healthcare availability. Choko et al. [34] and Knight et al. [52] found lower HIVST interest in rural or underserved areas due to infrastructure barriers and community stigma, whereas adults in urban areas with better healthcare access had higher acceptability. These findings highlight the need for targeted interventions to improve accessibility and reduce stigma, especially in conservative or resource-limited regions.

### Acceptability Rate

The review shows generally high acceptability of HIVST across populations, highlighting its potential in HIV prevention frameworks. Acceptance rates vary, with Agada et al. [32] reporting 23.4% acceptance, and Babatunde et al. [33] finding 62.6% willingness among students for future use. Key populations, such as MSM and FSWs, show strong interest in HIVST, with very high acceptability reported by Dirisu et al. [47] and Boisvert et al. [53], emphasizing its suitability for these groups. While some, like Oduetse et al. [43], report skepticism regarding user support, overall evidence favors HIVST as an accessible alternative to clinic-based testing.

### Ease of Use (Usability) and Perceived Need

Evidence shows that ease of use and perceived need significantly drive HIVST acceptability, especially among young adults in urban areas and FSWs. McHugh et al. [25] and Boisvert Moreau et al. [53] highlight that HIVST’s convenience and privacy appeal to high-risk groups who might avoid traditional testing due to logistical barriers or stigma. For MSM, ease of use is also favorable, though acceptance depends on privacy and stigma mitigation, as noted by Dirisu et al. [47] and Knox et al. [38]. Knight et al. [52] found that in general adult populations, acceptability hinged on usability and the perceived need for privacy and autonomy.

### Willingness to Pay

Although willingness to use HIVST was high, willingness to pay for kits varied widely, influenced by economic and access factors. Obiezu-Umeh et al. [42] and McHugh et al. [25] found that young adults often viewed cost as a barrier, underscoring the impact of affordability on acceptability. These findings suggest the need for affordable HIVST kits and subsidy models, especially in resource-limited settings, to enhance accessibility.

### User Errors in HIVST Implementation

User errors present a barrier to HIVST accuracy and effectiveness across priority populations. Choko et al. [34] found that although 98.5% rated the test as easy, 10% made minor errors, with another 10% needing assistance. Dirisu et al. [47] noted that MSM participants struggled with instructions, raising concerns about test accuracy without support. Among FSWs, Shava et al. [45] observed comprehension challenges due to literacy barriers, while McGowan et al. [40] emphasized the importance of peer-delivered instructional support for adolescent girls and young women (AGYW). These findings underscore the need for improved instructional materials to enhance correct test use, especially for first-time users.

### Implications for Targeted HIVST Outreach

These findings highlight the need for tailored HIVST outreach to meet each group’s unique needs. For young adults and FSWs, emphasizing HIVST’s privacy and stigma-free nature can sustain high acceptability. MSM populations require added confidentiality assurances and culturally sensitive education to reduce discrimination concerns. Addressing affordability and access issues is also essential to boost acceptability among general adult populations, especially in rural or conservative areas.

### Factors Influencing HIVST Uptake (Barriers and Facilitators)

Economic constraints emerged as a pervasive barrier to HIVST uptake across populations, particularly regarding the affordability of test kits for users. Studies by Obiezu-Umeh et al. [42] and McHugh et al. [25] highlight the financial burden on young adults seeking HIVST kits. Knight et al. [52] further noted that the availability of HIV self-test kits and their affordability were crucial determinants of acceptability among general adult populations. The study emphasized that individuals were more likely to use HIVST when kits were easily accessible through convenient distribution channels and when their cost was not a financial burden. Agada et al. [32] specifically identified the high cost of HIVST kits (1,700 naira, ∼$4.5) as a major barrier to uptake in Nigeria. The study emphasized that low-income individuals struggle to afford self-test kits, making free or subsidized distribution critical for expanding HIVST adoption. Similarly, Hatzold et al. [36], a study across Malawi and Zambia found that affordability remains one of the top concerns limiting HIVST uptake. Additionally, Babatunde et al. [33] reported that the cost of purchasing an HIVST kit was a significant determinant of willingness to self-test among Nigerian adolescents.

Stigma emerged as a significant barrier, especially for MSM and FSWs. Dirisu et al. [47] noted that MSM hesitated to use HIVST due to privacy concerns and fear of societal judgment. Similarly, Shava et al. [45] found that FSWs favored HIVST for its privacy, allowing them to avoid the stigma of public health settings. Knox et al. [29] observed that MSM acceptability varied based on healthcare access and social attitudes, emphasizing the need for culturally sensitive outreach and supportive environments to encourage testing.

Healthcare access also played a role in acceptability, with studies on general adult populations underscoring the challenges faced by those in underserved areas. For example, Choko et al. [34] and Knight et al. [52] found that limited healthcare infrastructure contributed to hesitation among general adult populations, particularly in rural or conservative communities where stigma and lack of resources intersect.

The privacy and autonomy of HIVST were widely valued, significantly facilitating its uptake across priority groups. McHugh et al. [25] found high acceptability among young adults, who appreciated the ability to test privately, reducing stigma. Boisvert Moreau et al. [53] reported similar positive responses from FSWs, who favored the self-administered nature of HIVST to avoid judgment. Obiezu-Umeh et al. [42] also noted that young adults valued privacy, bypassing the stigma associated with traditional testing facilities. However, limited post-test counseling, was highlighted by Dirisu et al. [47] Koris et al. [50] and Obiezu-Umeh et al. [42] remains a barrier, suggesting the need for linking HIVST with post-test support services to enhance uptake.

### Effectiveness of Distribution Strategies

The reviewed studies examined a variety of HIVST distribution strategies, including community-based, peer-led, and online methods. Peer-led approaches noted in studies involving MSM [47] and young adults [50], showed the potential to enhance HIVST uptake by leveraging trust within social networks. Community-based interventions, which facilitate greater privacy, were effective in reducing stigma and increasing uptake among FSWs by Kumwenda et al. [51] Online and digital platforms were also explored by Iwelunmor et al. [49] although further large-scale testing is needed to validate their effectiveness in increasing HIVST adoption across diverse populations.

### Insights From Quantitative, Qualitative, and Mixed-Methods Studies

Quantitative studies provided substantial evidence of high acceptability among certain populations, with more than 70% of respondents across FSWs and young adults indicating a willingness to use HIVST [25, 45, 50, 53]. Qualitative studies highlighted the personal and social factors driving this willingness, emphasizing the role of stigma avoidance and the perceived privacy of self-testing in encouraging uptake. Mixed-methods research corroborated these findings, offering a comprehensive insight into the contextual and demographic variations affecting acceptability [42, 47].

### Summary of Study Recommendations

The studies recommend tailored interventions to meet each group’s unique needs. Key strategies include leveraging peer and community support to reduce stigma, particularly for MSM and FSWs, and launching community-led educational campaigns to boost HIVST awareness among young adults and high-risk groups. Subsidy programs are advised to enhance HIVST access in economically disadvantaged communities. Furthermore, linking HIVST with post-test counseling and care services is essential for maximizing its public health impact in SSA.

## Discussion

This comprehensive review underscores the significant potential of HIVST in SSA to bridge critical gaps in HIV prevention and care. Across priority populations—including FSWs, young adults, MSM, and general adult populations—the findings suggest a high level of acceptability for HIVST due to factors such as privacy [23, 34, 39, 47], autonomy [46, 50, 52], empowerment [48] and ease of use [26, 53, 59]. This high acceptability underscores HIVST’s potential as a transformative tool for increasing HIV testing rates in SSA, where barriers to traditional testing methods persist.

Our review shows that HIVST acceptability varies significantly by population group, influenced by stigma, healthcare access, and socio-cultural factors. For FSWs and young adults, the private nature of HIVST was a significant facilitator, allowing them to avoid the stigma associated with public health facilities [42, 45]. However, MSM populations displayed more variable acceptance, with privacy concerns and fear of discrimination acting as barriers to uptake [38, 47]. While HIVST reduces the stigma associated with facility-based testing, concerns remain regarding the visibility of kit collection, the confidentiality of supervised self-testing, and post-test linkage to care. Dirisu et al. [47] noted that MSM preferred anonymous or peer-based distribution channels but feared that obtaining kits from known individuals might lead to unintentional disclosure. General adult populations in rural or underserved areas also exhibited moderate acceptability, linked to limited healthcare resources and the perceived social consequences of HIV testing [34, 52]. These responses underscore the need for targeted outreach strategies that address population-specific concerns, ensuring tailored messaging that speaks to each group’s unique experiences with stigma, privacy, and healthcare access.

### Economic Barriers and the Need for Cost Subsidization

Economic constraints emerged as a prevalent barrier to HIVST uptake across populations, particularly regarding the affordability of test kits for users. Studies indicate that MSM and young adults often struggle with the financial burden of purchasing self-test kits, limiting their ability to test regularly [13, 42, 58]. This financial barrier is particularly relevant in resource-limited settings within SSA, where cost considerations may discourage routine HIV testing and exacerbate existing inequities in access. Studies by Obiezu-Umeh et al. [42] and McHugh et al. [25] highlight that young adults face affordability concerns when seeking HIVST kits, reinforcing the need for cost-effective solutions. Similarly, Knight et al. [52] found that the availability and affordability of HIVST kits were crucial determinants of acceptability among general adult populations. Individuals were more likely to adopt HIVST when test kits were easily accessible through convenient distribution channels and when their cost was not a financial burden. Beyond broad affordability concerns, Agada et al. [32] specifically identified the high cost of HIVST kits as a major barrier to uptake in Nigeria. The study emphasized that low-income individuals struggle to afford self-test kits, making free or subsidized distribution critical for expanding HIVST adoption, particularly among vulnerable groups. Likewise, Hatzold et al. [36], who found that affordability remains one of the most significant concerns limiting HIVST uptake. Babatunde et al. [33] further highlighted that the cost of purchasing an HIVST kit significantly influenced willingness to self-test among Nigerian adolescents, emphasizing how economic constraints directly impact HIV prevention efforts among young populations. Addressing these economic challenges is crucial for the sustainability and scalability of HIVST in SSA, where resource allocation and budget constraints remain pressing concerns. Cost-subsidized or free HIVST kits, as suggested by Iliyasu et al. [37], Knight et al. [52], and Ben Moussa et al. [26], could mitigate these barriers, enabling broader access and uptake, especially among economically disadvantaged groups. Additionally, integrating cost-effective distribution models, such as community-based distribution and social marketing strategies, may help ensure equitable access to HIVST while reducing financial barriers to testing.

### Privacy as a Key Facilitator Across Populations

Privacy and autonomy consistently emerged as facilitators of HIVST, particularly among high-risk groups like FSWs and MSM, who face higher levels of stigma in traditional testing environments [39, 41, 47, 51, 53, 54]. The self-administered nature of HIVST enables these groups to bypass public testing facilities, offering a more private and less judgmental avenue for testing [45]. For young adults, privacy not only encourages uptake but also fosters a sense of empowerment, as they can engage in health-seeking behavior without disclosing their HIV status to others [25, 50]. However, privacy alone may not suffice; integrating HIVST with post-test support services, such as counseling and linkage to care, could enhance the utility and effectiveness of HIVST, addressing unmet needs in follow-up care and support [32, 47, 48].

### Varied Acceptability in MSM and the Role of Culturally Sensitive Outreach

While HIVST acceptability was generally high, MSM populations exhibited variability in response, affected by privacy concerns and perceived stigma [23, 47, 54, 55]. A study conducted among young men who have sex with men aged 19–30 years in Uganda by Okoboi et al. [27] identified several factors influencing HIVST acceptability, including efficiency, confidentiality, non-invasiveness, reduced stigma, and peer support networks. These findings highlight the importance of culturally sensitive approaches in HIVST implementation. These findings indicate that for MSM, additional assurances of confidentiality and culturally sensitive outreach are essential to promote HIVST uptake. Educational campaigns specifically tailored to MSM, which address stigma and reinforce confidentiality protections, could play a critical role in increasing HIVST use within this population. Moreover, engaging MSM-led organizations and trusted community figures in HIVST awareness campaigns may help alleviate some of the stigma associated with testing, creating a safer and more supportive environment.

### User Errors as a Barrier to HIVST

This review identifies user errors as a critical barrier impacting the accuracy and reliability of HIVST across priority populations in SSA. Minor procedural mistakes, as observed in Choko et al. [34] where 10% of participants reported difficulties with the test process despite an overall positive ease-of-use rating, indicate the need for more intuitive instructional materials. Similarly, MSM participants in Dirisu et al. [47] noted issues with comprehending test instructions, suggesting that complex procedures may undermine self-testing confidence when direct support is unavailable. Among FSWs, Shava et al. [45] found that low literacy levels exacerbated concerns over self-competency and accuracy, highlighting the need for accessible and user-friendly guides. Additionally, McGowan et al. [40] demonstrated that peer-delivered models for AGYW engaging in Pre-Exposure Prophylaxis (PrEP) could provide the instructional support necessary to mitigate errors, thus reinforcing the value of guided assistance.

### Effectiveness of Distribution Strategies and the Importance of Community Engagement

This review highlights promising HIVST distribution strategies, including peer-led [25, 40, 51], community-based [36, 53], and digital approaches [23] which show the potential to reach diverse populations. Peer-led initiatives, particularly among men, young adults, MSM, and FSWs effectively leverage social networks to foster trust and encourage HIVST adoption [49, 53, 54]. Community-based distribution, which emphasizes local involvement and reduces stigma through trusted networks, has shown particular efficacy among FSWs [51]. However, while these methods are promising, they require rigorous evaluation to assess long-term effectiveness, particularly in areas where healthcare resources and follow-up care are limited.

### Addressing the Need for Subsidies and Post-Test Counseling

The findings underscore the critical need for subsidized HIVST kits to ensure accessibility for economically disadvantaged groups, as well as integrated post-test counseling to support individuals who may require additional healthcare guidance [37, 42, 45]. Linkage to care remains a vital yet underexplored component of HIVST, particularly for those who test positive and need prompt medical intervention.

### Implications for Public Health Strategies

The high acceptability of HIVST among priority populations suggests that this approach could significantly contribute to achieving UNAIDS’ 95–95–95 targets by increasing the number of individuals who know their HIV status. However, to realize HIVST’s full potential, public health strategies must consider the distinct barriers and facilitators identified across demographic groups. By addressing economic, social, and cultural factors and by implementing scalable distribution strategies that leverage community resources, SSA can enhance HIVST adoption and extend HIV prevention efforts to underserved populations.

### Limitations of the Review

Our review has several limitations. The exclusion of non-English studies may have led to the omission of relevant findings from SSA’s diverse linguistic regions. Furthermore, this review did not include a formal quality assessment, as the primary aim of a scoping review is to map the existing literature rather than critically evaluate the quality of individual studies. While formal quality appraisal is not typically required for scoping reviews, its absence may limit the ability to assess the robustness of the included evidence. We recommend that future systematic reviews incorporate rigorous quality assessments to strengthen the evaluation of study reliability and enhance their implications for policy and practice [64]. The heterogeneity of study designs and population groups included also presents challenges in comparing findings directly, underscoring the importance of standardized research approaches in future HIVST studies.

Another key limitation of this review is that while it synthesizes evidence on HIV self-testing acceptability and uptake, many included studies do not consistently report details on the type of self-test (oral-fluid vs. blood-based) or the testing supervision model (supervised vs. unsupervised). This variability limits our ability to analyze whether these distinctions influence acceptability rates or uptake. Future research should aim to provide more granular reporting on HIVST modalities to better understand how different self-testing methods and implementation strategies impact uptake among diverse populations.

### Conclusion

HIV self-testing holds significant promise as a tool for expanding HIV testing access across priority populations in SSA. Our findings indicate that while HIVST enjoys broad acceptability, its successful implementation is contingent on addressing economic barriers, ensuring confidentiality, and providing linkage to care services. Strategic investment, supportive policy, and culturally sensitive outreach are essential to integrate HIVST effectively into SSA’s HIV prevention frameworks. Further research should focus on evaluating HIVST’s cost-effectiveness, sustainability, and impact on long-term health outcomes, enabling a more robust response to the HIV epidemic in SSA.

## Abbreviations

AGYW, Adolescent Girls and Young Women; FSWs, Female Sex Workers; HIVST, HIV Self-Testing; KPs, Key populations; MSM, Men Who Have Sex with Men; PIOT, Population-Intervention-Outcome-Timeline; PPs, Priority Populations; PrEP, Pre-Exposure Prophylaxis; PRISMA-ScR, Preferred Reporting Items for Systematic Reviews and Meta-Analyses Extension for Scoping Reviews; PWID, people who inject drugs (PWID); SSA, Sub-Saharan Africa; UNAIDS, Joint United Nations Programme on HIV/AIDS.
