# Supplementary material for: Factors Influencing the Acceptability and Uptake of HIV Self-Testing Among Priority Populations in Sub-Saharan Africa: A Scoping Review
Source: Public Health Rev. 2025 Apr 22;46:1608140. doi: 10.3389/phrs.2025.1608140 (PMC12052600; doi:10.3389/phrs.2025.1608140)
Supplement: Supplementary file 2 [file Table1.docx]

Stage 1: Core Concepts - HIV, Self-Testing, and SSA Region

("HIV"[MeSH Terms] AND ("Self-Testing"[MeSH Terms] OR "HIV Testing"[MeSH Terms]) AND ("Africa South of the Sahara"[MeSH Terms] OR "Sub-Saharan Africa" [All Fields])) OR ("HIV"[MeSH Terms] AND ("Self-Testing"[MeSH Terms] OR "HIV Testing"[MeSH Terms])

Stage 2: Specific Populations - Priority and Vulnerable Populations

("Vulnerable Populations"[MeSH Terms] OR "Priority Populations"[All Fields] OR "Sexual and Gender Minorities"[MeSH Terms] OR "Sex Workers"[MeSH Terms] OR "Working Poor"[MeSH Terms] OR "Poverty"[MeSH Terms] OR "Adolescents"[MeSH Terms] OR "Young Adults"[MeSH Terms]))

Stage 3: Date Range and Additional Filters

("2010"[Date - Publication] : "2023"[Date - Publication])

Stage 4: Final Combination of Core and Specific Stages

("HIV"[MeSH Terms] AND ("Self-Testing"[MeSH Terms] OR "HIV Testing"[MeSH Terms]) AND ("Africa South of the Sahara"[MeSH Terms] OR "Sub-Saharan Africa" [All Fields])) OR ("HIV"[MeSH Terms] AND ("Self-Testing"[MeSH Terms] OR "HIV Testing"[MeSH Terms]) AND ("Vulnerable Populations"[MeSH Terms] OR "Priority Populations"[All Fields] OR "Sexual and Gender Minorities"[MeSH Terms] OR "Sex Workers"[MeSH Terms] OR "Working Poor"[MeSH Terms] OR "Poverty"[MeSH Terms] OR "Adolescents"[MeSH Terms] OR "Young Adults"[MeSH Terms])) AND ("2010"[Date - Publication] : "2023"[Date - Publication])
